# Supplementary material for: Exosomes from bone marrow mesenchymal stem cells protect melanocytes under vitiligo-related conditions through induction of NRF2/HO1 expression
Source: PLoS One. 2025 Dec 4;20(12):e0338323. doi: 10.1371/journal.pone.0338323 (PMC12677447; doi:10.1371/journal.pone.0338323)
Supplement: S1 Table — (DOCX) [file pone.0338323.s001.docx]

### **Table S1. Experimental groups and treatments in PIG3V cells**

| **Group** | **Description** |
| --- | --- |
| **Ctrl** | Untreated PIG3V cells |
| **BMSCs-medium** | PIG3V cells were treated with BMSCs-conditioned medium only |
| **BMSCs-GW4869** | PIG3V cells were treated with medium from BMSCs pretreated with exosome inhibitor GW4869 |
| **BMSCs-Exos** | PIG3V cells were treated with BMSCs-derived exosomes |
| **H₂O₂** | PIG3V cells were exposed to 100 µM H₂O₂ for 2 h and then cultured under normal conditions |
| **BMSCs-medium + H₂O₂** | PIG3V cells were treated with BMSCs-conditioned medium and exposed to 100 µM H₂O₂ for 2 h, followed by normal culture |
| **BMSCs-GW4869 + H₂O₂** | PIG3V cells were treated with medium from BMSCs pretreated with exosome inhibitor GW4869 and exposed to 100 µM H₂O₂ for 2 h, followed by normal culture |
| **BMSCs-Exos + H₂O₂** | PIG3V cells were treated with BMSCs-derived exosomes and exposed to 100 µM H₂O₂ for 2 h, followed by normal culture |
