# Supplementary material for: Exosomes from bone marrow mesenchymal stem cells protect melanocytes under vitiligo-related conditions through induction of NRF2/HO1 expression
Source: PLoS One. 2025 Dec 4;20(12):e0338323. doi: 10.1371/journal.pone.0338323 (PMC12677447; doi:10.1371/journal.pone.0338323)
Supplement: S2 Table — (DOCX) [file pone.0338323.s002.docx]

### **Table S2. Experimental groups and treatment in PIG3V cells**

| **Group** | **Experimental description** |
| --- | --- |
| **NC siRNA** | PIG3V cells were transfected with NC siRNA and cultured under normal conditions |
| **NRF2 siRNA** | PIG3V cells were transfected with *NRF2* siRNA and cultured under normal conditions |
| **NC siRNA + BMSCs-Exos** | PIG3V cells were transfected with NC siRNA and treated with BMSCs-Exos, followed by normal culture |
| **NRF2 siRNA + BMSCs-Exos** | PIG3V cells were transfected with *NRF2* siRNA and treated with BMSCs-Exos, followed by normal culture |
| **NC siRNA + H₂O₂** | PIG3V cells were transfected with NC siRNA and exposed to 100 µM H₂O₂ for 2 h, followed by normal culture |
| **NRF2 siRNA + H₂O₂** | PIG3V cells were transfected with *NRF2* siRNA and exposed to 100 µM H₂O₂ for 2 h, followed by normal culture |
| **NC siRNA + BMSCs-Exos + H₂O₂** | PIG3V cells were transfected with NC siRNA and treated with BMSCs-Exos, followed by exposure to 100 µM H₂O₂ for 2 h and subsequent normal culture |
| **NRF2 siRNA + BMSCs-Exos + H₂O₂** | PIG3V cells were transfected with *NRF2* siRNA and treated with BMSCs-Exos, followed by exposure to 100 µM H₂O₂ for 2 h and subsequent normal culture |
