# Supplementary figures and images for: Exosomes from bone marrow mesenchymal stem cells protect melanocytes under vitiligo-related conditions through induction of NRF2/HO1 expression
Source: PLoS One. 2025 Dec 4;20(12):e0338323. doi: 10.1371/journal.pone.0338323 (PMC12677447; doi:10.1371/journal.pone.0338323)

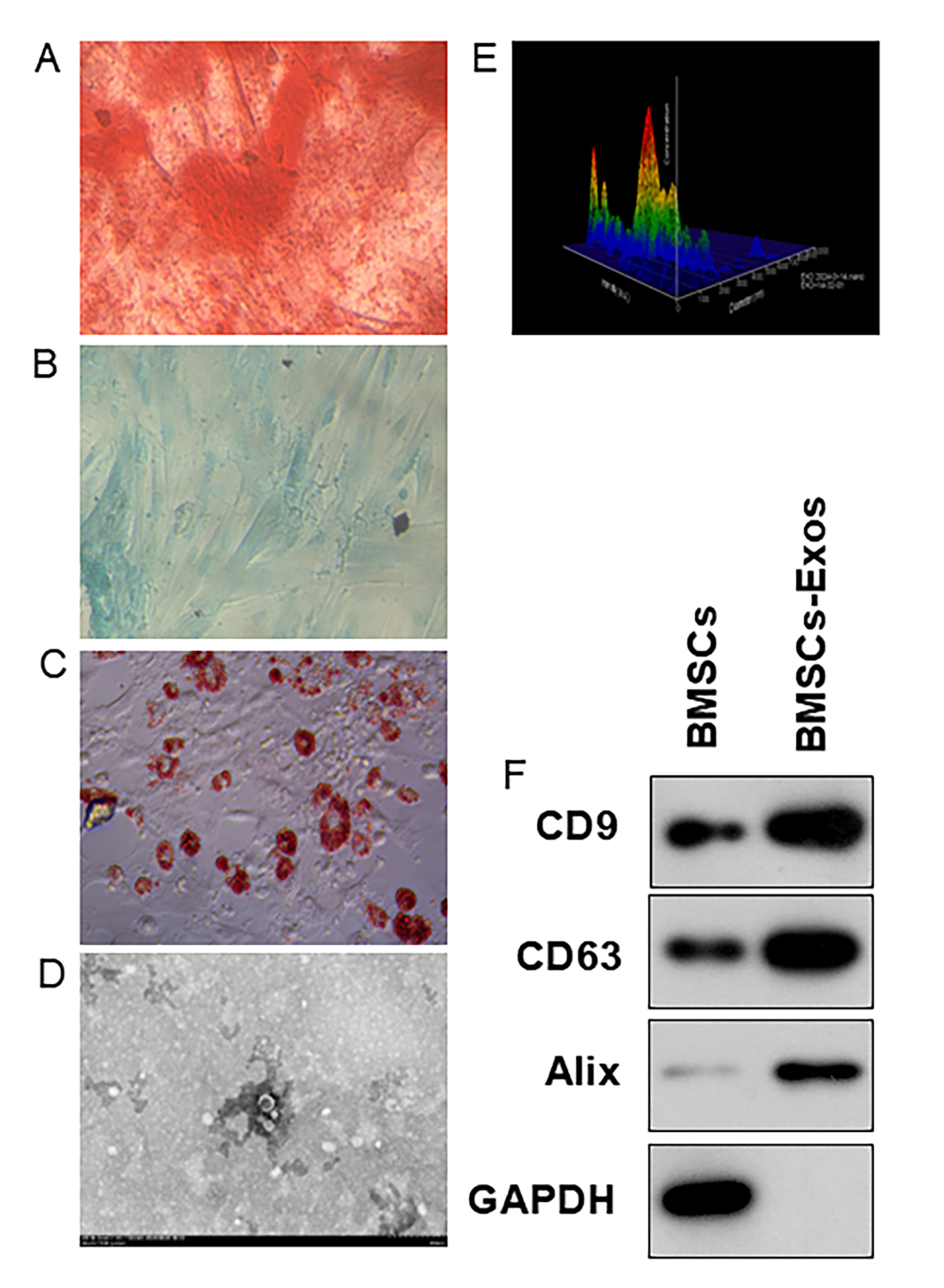

Supplement: S1 Fig — BMSCs were differentiated in (A) osteogenic medium for 21 days and stained with Alizarin Red S (X100), (B) adipogenic medium for 14 days and stained with Oil Red O (X100), and (C) chondrogenic medium for 21 days and stained with Alcian Blue (X100). Isolated BMSC-Exos were identified by TEM (X4000, D), NTA analysis (E), and Western blot analysis for exosomal markers CD9, CD63, and Alix, with GAPDH as internal control (F). (TIF) [file pone.0338323.s003.tif]
