# Supplementary material for: Exosomes from bone marrow mesenchymal stem cells protect melanocytes under vitiligo-related conditions through induction of NRF2/HO1 expression
Source: PLoS One. 2025 Dec 4;20(12):e0338323. doi: 10.1371/journal.pone.0338323 (PMC12677447; doi:10.1371/journal.pone.0338323)
Supplement: S1 File — (DOCX) [file pone.0338323.s004.docx]

**Table-Fig 2A Raw data for Fig 2A**

|  | Ctrl | BMSCs-medium | BMSCs-GW4869 | BMSCs-Exos | H_2_O_2_ | BMSCs-medium  +H_2_O_2_ | BMSCs-GW4869  +H_2_O_2_ | BMSC-Exos  +H_2_O_2_ |
| --- | --- | --- | --- | --- | --- | --- | --- | --- |
| 0h Repeat 1 | 92.71 | 95.46 | 93.80 | 93.70 | 107.84 | 114.74 | 105.58 | 101.20 |
| Repeat 2 | 105.62 | 85.61 | 103.88 | 96.77 | 95.02 | 91.19 | 113.12 | 106.45 |
| Repeat 3 | 97.26 | 102.27 | 100.78 | 99.08 | 122.17 | 91.19 | 107.84 | 89.21 |
| Repeat 4 | 95.75 | 114.39 | 96.12 | 100.614 | 86.727 | 97.26 | 85.97 | 92.95 |
| Repeat 5 | 108.66 | 102.27 | 105.43 | 109.831 | 88.235 | 105.62 | 87.48 | 110.20 |
| Mean | 100.00 | 100.00 | 100.00 | 100.00 | 100 | 100.00 | 100.00 | 100.00 |
| Standard deviations | 6.81 | 10.55 | 4.96 | 6.08 | 14.94 | 10.15 | 12.43 | 8.85 |
| 24h Repeat 1 | 150.46 | 159.10 | 151.16 | 168.97 | 107.09 | 129.94 | 128.96 | 160.42 |
| Repeat 2 | 141.34 | 161.36 | 124.81 | 160.52 | 126.70 | 120.82 | 104.83 | 118.44 |
| Repeat 3 | 132.22 | 126.52 | 154.26 | 155.15 | 98.79 | 123.10 | 105.58 | 131.93 |
| Repeat 4 | 128.42 | 157.58 | 127.13 | 160.52 | 106.34 | 122.34 | 135.5 | 122.94 |
| Repeat 5 | 120.06 | 123.49 | 124.03 | 165.90 | 124.43 | 124.62 | 117.65 | 152.17 |
| Mean | 134.50 | 145.61 | 136.28 | 162.21 | 112.67 | 124.16 | 118.55 | 137.18 |
| Standard deviations | 11.75 | 18.89 | 15.09 | 5.36 | 12.24 | 3.51 | 13.80 | 18.35 |
| 48h Repeat 1 | 528.12 | 656.06 | 478.30 | 658.22 | 194.57 | 265.96 | 249.62 | 544.98 |
| Repeat 2 | 462.77 | 595.46 | 577.52 | 574.50 | 208.15 | 309.27 | 239.82 | 494.75 |
| Repeat 3 | 560.03 | 568.94 | 626.36 | 591.40 | 219.46 | 273.56 | 234.54 | 529.24 |
| Repeat 4 | 504.56 | 587.12 | 531.01 | 698.16 | 196.08 | 291.79 | 243.59 | 554.72 |
| Repeat 5 | 463.53 | 573.49 | 528.68 | 610.60 | 169.683 | 303.95 | 256.41 | 606.45 |
| Mean | 503.80 | 596.21 | 548.37 | 626.57 | 197.59 | 288.91 | 244.80 | 546.03 |
| Standard deviations | 42.01 | 35.09 | 55.97 | 50.82 | 18.57 | 18.79 | 8.51 | 40.74 |
| 72h Repeat 1 | 773.56 | 1016.67 | 803.88 | 1127.50 | 350.68 | 575.23 | 468.33 | 887.56 |
| Repeat 2 | 764.44 | 900.00 | 905.43 | 1164.36 | 390.65 | 438.45 | 420.81 | 874.81 |
| Repeat 3 | 771.28 | 974.24 | 857.36 | 1225.81 | 271.49 | 481.76 | 369.53 | 844.08 |
| Repeat 4 | 792.55 | 861.36 | 943.41 | 1112.14 | 312.22 | 530.40 | 460.03 | 840.33 |
| Repeat 5 | 705.93 | 915.15 | 927.13 | 1166.67 | 446.46 | 501.52 | 478.88 | 829.09 |
| Mean | 761.55 | 933.49 | 887.44 | 1159.29 | 354.30 | 505.47 | 439.52 | 855.17 |
| Standard deviations | 32.80 | 61.72 | 56.83 | 43.99 | 67.92 | 51.37 | 44.86 | 24.79 |
| 96h Repeat 1 | 784.95 | 1293.94 | 1121.71 | 1370.97 | 558.82 | 772.04 | 397.44 | 1023.24 |
| Repeat 2 | 889.06 | 1169.70 | 1242.64 | 1515.36 | 464.56 | 600.30 | 601.06 | 925.04 |
| Repeat 3 | 1069.91 | 1135.61 | 946.51 | 1548.39 | 402.72 | 687.7 | 594.27 | 1194.90 |
| Repeat 4 | 846.51 | 1027.27 | 1139.54 | 1295.70 | 284.31 | 512.16 | 606.34 | 1041.23 |
| Repeat 5 | 926.29 | 1162.12 | 1164.34 | 1244.24 | 365.76 | 757.60 | 542.99 | 1122.19 |
| Mean | 903.34 | 1157.73 | 1122.95 | 1394.93 | 415.23 | 665.96 | 548.42 | 1061.32 |
| Standard deviations | 106.91 | 95.16 | 108.91 | 133.39 | 103.43 | 109.64 | 88.11 | 102.44 |

**Table-Fig 2B Raw data for Fig 2**

|  | Ctrl | BMSCs-medium | BMSCs-GW4869 | BMSCs-Exos | H_2_O_2_ | BMSCs-medium  +H_2_O_2_ | BMSCs-GW4869  +H_2_O_2_ | BMSCs-Exos  +H_2_O_2_ |
| --- | --- | --- | --- | --- | --- | --- | --- | --- |
| Repeat 1 | 784.95 | 1293.94 | 1121.71 | 1370.97 | 558.82 | 772.04 | 397.44 | 1023.24 |
| Repeat 2 | 889.06 | 1169.70 | 1242.64 | 1515.36 | 464.56 | 600.30 | 601.06 | 925.04 |
| Repeat 3 | 1069.91 | 1135.61 | 946.51 | 1548.39 | 402.72 | 687.69 | 594.27 | 1194.90 |
| Repeat 4 | 846.51 | 1027.27 | 1139.54 | 1295.70 | 284.31 | 512.16 | 606.34 | 1041.23 |
| Repeat 5 | 926.29 | 1162.12 | 1164.34 | 1244.24 | 365.76 | 757.60 | 542.99 | 1122.19 |
| Mean | 903.34 | 1157.73 | 1122.95 | 1394.93 | 415.23 | 665.96 | 548.42 | 1061.32 |
| Standard deviations | 106.91 | 95.16 | 108.91 | 133.40 | 103.43 | 109.64 | 88.11 | 102.44 |

**Table-Fig 2C Raw data for Fig 2C**

|  | Ctrl | BMSCs-medium | BMSCs-GW4869 | BMSCs-Exos | H_2_O_2_ | BMSCs-medium  +H_2_O_2_ | BMSCs-GW4869  +H_2_O_2_ | BMSCs-Exos  +H_2_O_2_ |
| --- | --- | --- | --- | --- | --- | --- | --- | --- |
| Repeat 1 | 8.78 | 6.42 | 7.34 | 3.78 | 23.12 | 14.51 | 16.85 | 9.06 |
| Repeat 2 | 9.41 | 5.98 | 6.85 | 4.06 | 23.16 | 13.54 | 16.97 | 8.98 |
| Repeat 3 | 8.77 | 6.57 | 6.99 | 3.61 | 22.44 | 14.42 | 17.31 | 9.05 |
| Mean | 8.99 | 6.32 | 7.06 | 3.82 | 22.91 | 14.16 | 17.05 | 9.03 |
| Standard deviations | 0.36 | 0.31 | 0.25 | 0.23 | 0.40 | 0.54 | 0.24 | 0.044 |

**Table-Fig 3A Raw data for Fig 3A**

|  | Ctrl | BMSCs-medium | BMSCs-GW4869 | BMSCs-Exos | H_2_O_2_ | BMSCs-medium  +H_2_O_2_ | BMSCs-GW4869  +H_2_O_2_ | BMSsC-Exos  +H_2_O_2_ |
| --- | --- | --- | --- | --- | --- | --- | --- | --- |
| Repeat 1 | 2.88 | 3.67 | 2.99 | 5.49 | 1.29 | 1.97 | 1.61 | 2.99 |
| Repeat 2 | 2.33 | 4.15 | 3.67 | 4.29 | 1.18 | 2.39 | 1.16 | 3.78 |
| Repeat 3 | 2.67 | 4.09 | 2.89 | 5.93 | 1.55 | 1.93 | 1.93 | 2.57 |
| Mean | 2.63 | 3.97 | 3.18 | 5.24 | 1.34 | 2.10 | 1.57 | 3.11 |
| Standard deviations | 0.278 | 0.26 | 0.42 | 0.85 | 0.19 | 0.25 | 0.39 | 0.61 |

**Table-Fig 3B Raw data for Fig 3B**

|  | Ctrl | BMSCs-medium | BMSCs-GW4869 | BMSCs-Exos | H_2_O_2_ | BMSCs-medium  +H_2_O_2_ | BMSCs-GW4869  +H_2_O_2_ | BMSCs-Exos  +H_2_O_2_ |
| --- | --- | --- | --- | --- | --- | --- | --- | --- |
| Repeat 1 | 1.55 | 1.61 | 1.28 | 0.89 | 3.44 | 2.64 | 3.10 | 1.76 |
| Repeat 2 | 1.72 | 1.27 | 1.55 | 0.62 | 3.62 | 2.29 | 3.30 | 2.00 |
| Repeat 3 | 1.94 | 1.11 | 1.76 | 1.08 | 3.22 | 2.79 | 2.98 | 1.45 |
| Mean | 1.74 | 1.33 | 1.53 | 0.86 | 3.43 | 2.57 | 3.13 | 1.74 |
| Standard deviations | 0.278 | 0.26 | 0.42 | 0.85 | 0.19 | 0.25 | 0.39 | 0.61 |

**Table-Fig 3C Raw data for Fig 3C**

|  | Ctrl | BMSCs-medium | BMSCs-GW4869 | BMSCs-Exos | H_2_O_2_ | BMSCs-medium  +H_2_O_2_ | BMSCs-GW4869  +H_2_O_2_ | BMSCs-Exos  +H_2_O_2_ |
| --- | --- | --- | --- | --- | --- | --- | --- | --- |
| Repeat 1 | 3.76 | 5.49 | 4.41 | 7.22 | 1.65 | 3.05 | 2.27 | 4.29 |
| Repeat 2 | 3.43 | 5.34 | 4.09 | 6.99 | 1.9 | 2.8 | 2.11 | 4.38 |
| Repeat 3 | 3.56 | 5.22 | 4.26 | 7.2 | 1.71 | 2.82 | 1.96 | 4.18 |
| Mean | 3.58 | 5.35 | 4.25 | 7.14 | 1.75 | 2.89 | 2.11 | 4.28 |
| Standard deviations | 0.17 | 0.14 | 0.16 | 0.13 | 0.13 | 0.14 | 0.16 | 0.10 |

**Table-Fig 3D Raw data for Fig 3D**

|  | Ctrl | BMSCs-medium | BMSCs-GW4869 | BMSCs-Exos | H_2_O_2_ | BMSCs-medium  +H_2_O_2_ | BMSCs-GW4869  +H_2_O_2_ | BMSCs-Exos  +H_2_O_2_ |
| --- | --- | --- | --- | --- | --- | --- | --- | --- |
| Repeat 1 | 0.99 | 0.68 | 0.74 | 0.45 | 2.22 | 1.36 | 1.80 | 0.99 |
| Repeat 2 | 1.02 | 0.67 | 0.71 | 0.46 | 2.24 | 1.37 | 1.75 | 1.00 |
| Repeat 3 | 0.99 | 0.65 | 0.73 | 0.42 | 2.23 | 1.35 | 1.81 | 1.03 |
| Mean | 1.00 | 0.67 | 0.73 | 0.44 | 2.23 | 1.36 | 1.79 | 1.00 |
| Standard deviations | 0.02 | 0.014 | 0.017 | 0.02 | 0.010 | 0.0083 | 0.033 | 0.021 |

**Table-Fig 4 Raw data for Fig 4**

|  |  | Ctrl | BMSCs-medium | BMSCs-GW4869 | BMSCs-Exos | Ctrl+H_2_O_2_ | BMSCs-medium  +H_2_O_2_ | BMSCs-GW4869  +H_2_O_2_ | BMSCs-Exos  +H_2_O_2_ |
| --- | --- | --- | --- | --- | --- | --- | --- | --- | --- |
| KI67 | Repeat 1 | 1.04 | 1.36 | 0.81 | 1.65 | 0.22 | 0.68 | 0.22 | 1.34 |
|  | Repeat 2 | 0.99 | 1.4 | 0.79 | 1.62 | 0.24 | 0.65 | 0.23 | 1.26 |
|  | Repeat 3 | 0.97 | 1.4 | 0.77 | 1.63 | 0.22 | 0.65 | 0.23 | 1.30 |
|  | Mean | 1.00 | 1.39 | 0.79 | 1.63 | 0.23 | 0.66 | 0.23 | 1.30 |
|  | Standard deviations | 0.0361 | 0.0231 | 0.02 | 0.0153 | 0.0115 | 0.017 | 0.0058 | 0.04 |
| NRF2 | Repeat 1 | 1.07 | 2.67 | 1.28 | 2.84 | 0.22 | 0.71 | 0.16 | 2.23 |
|  | Repeat 2 | 0.94 | 2.63 | 1.39 | 2.78 | 0.25 | 0.67 | 0.18 | 2.12 |
|  | Repeat 3 | 0.99 | 2.60 | 1.32 | 2.71 | 0.24 | 0.65 | 0.18 | 2.16 |
|  | Mean | 1.00 | 2.63 | 1.33 | 2.78 | 0.24 | 0.68 | 017 | 2.17 |
|  | Standard deviations | 0.0656 | 0.0351 | 0.0557 | 0.0651 | 0.0153 | 0.0306 | 0.0115 | 0.0557 |
| HO1 | Repeat 1 | 0.97 | 1.28 | 0.87 | 1.38 | 0.28 | 0.76 | 0.43 | 1.45 |
|  | Repeat 2 | 1.03 | 1.29 | 0.95 | 1.44 | 0.35 | 0.75 | 0.44 | 1.47 |
|  | Repeat 3 | 1.00 | 1.33 | 0.93 | 1.44 | 0.36 | 0.71 | 0.46 | 1.40 |
|  | Mean | 1.00 | 1.30 | 0.92 | 1.42 | 0.33 | 0.74 | 0.443 | 1.44 |
|  | Standard deviations | 0.03 | 0.0265 | 0.0416 | 0.0346 | 0.0436 | 0.026 | 0.0153 | 0.0361 |
| BAX | Repeat 1 | 1.01 | 0.59 | 0.84 | 0.20 | 1.21 | 0.85 | 1.06 | 0.53 |
|  | Repeat 2 | 0.97 | 0.56 | 0.87 | 0.21 | 1.26 | 0.85 | 1.04 | 0.55 |
|  | Repeat 3 | 1.02 | 0.58 | 0.87 | 0.21 | 1.24 | 0.84 | 1.05 | 0.54 |
|  | Mean | 1 | 0.58 | 0.86 | 0.21 | 1.24 | 0.85 | 1.05 | 0.54 |
|  | Standard deviations | 0.0265 | 0.0153 | 0.0173 | 0.00577 | 0.0252 | 0.00577 | 0.01 | 0.01 |
| BCL-2 | Repeat 1 | 0.95 | 5.96 | 2.07 | 8.03 | 1.08 | 3.27 | 0.82 | 7.93 |
|  | Repeat 2 | 1.04 | 5.86 | 1.94 | 7.49 | 1.09 | 3.07 | 0.84 | 7.88 |
|  | Repeat 3 | 1.01 | 5.79 | 1.97 | 7.32 | 1.10 | 2.84 | 0.88 | 7.77 |
|  | Mean | 1.00 | 5.87 | 1.99 | 7.613 | 1.09 | 3.06 | 0.85 | 7.86 |
|  | Standard deviations | 0.0458 | 0.0854 | 0.0681 | 0.37 | 0.01 | 0.22 | 0.0306 | 0.0819 |

**Table-Fig 5A Raw data for Fig 5A**

|  | NC siRNA | Nrf2 siRNA | NC siRNA+  BMSCs-Exos | Nrf2 siRNA+  BMSCs-Exos | NC siRNA  +H_2_O_2_ | Nrf2 siRNA  +H_2_O_2_ | NC siRNA+  BMSCs-Exos+H_2_O_2_ | Nrf2 siRNA+  BMSCs-Exos+H_2_O_2_ |
| --- | --- | --- | --- | --- | --- | --- | --- | --- |
| 0h Repeat 1 | 148.20 | 122.70 | 167.28 | 172.44 | 142.40 | 126.54 | 150.43 | 130.11 |
| Repeat 2 | 161.72 | 147.84 | 160.22 | 152.95 | 123.93 | 116.89 | 148.02 | 138.87 |
| Repeat 3 | 128.34 | 140.54 | 154.73 | 162.70 | 116.70 | 111.26 | 137.58 | 154.01 |
| Repeat 4 | 147.40 | 121.89 | 180.61 | 177.32 | 125.54 | 110.56 | 134.37 | 119.76 |
| Repeat 5 | 138.65 | 127.57 | 177.47 | 174.88 | 121.52 | 128.95 | 138.38 | 136.48 |
| Mean | 144.86 | 132.11 | 168.06 | 168.06 | 126.02 | 118.82 | 141.76 | 135.85 |
| Standard deviations | 12.39 | 11.53 | 11.02 | 10.10 | 9.75 | 8.56 | 7.03 | 12.56 |
| 24h Repeat 1 | 148.20 | 122.70 | 167.28 | 172.44 | 142.40 | 126.54 | 150.43 | 130.11 |
| Repeat 2 | 161.72 | 147.84 | 160.22 | 152.95 | 123.93 | 116.89 | 148.02 | 138.87 |
| Repeat 3 | 128.31 | 140.54 | 154.73 | 162.70 | 116.70 | 111.26 | 137.58 | 154.01 |
| Repeat 4 | 147.40 | 121.89 | 180.61 | 177.32 | 125.54 | 110.46 | 134.37 | 119.76 |
| Repeat 5 | 138.65 | 127.57 | 177.47 | 174.88 | 121.52 | 128.95 | 138.38 | 136.48 |
| Mean | 144.86 | 132.10 | 168.06 | 168.06 | 126.02 | 118.82 | 141.76 | 135.85 |
| Standard deviations | 12.39 | 11.53 | 11.02 | 10.10 | 9.75 | 8.56 | 7.03 | 12.56 |
| 48h Repeat 1 | 460.76 | 444.60 | 593.05 | 648.35 | 286.14 | 222.25 | 460.39 | 397.77 |
| Repeat 2 | 425.77 | 421.08 | 546.79 | 524.91 | 191.38 | 190.08 | 472.43 | 426.45 |
| Repeat 3 | 498.14 | 364.32 | 593.83 | 505.41 | 212.26 | 150.67 | 445.13 | 439.99 |
| Repeat 4 | 435.31 | 374.05 | 694.20 | 528.97 | 185.76 | 170.78 | 396.95 | 335.64 |
| Repeat 5 | 523.60 | 325.41 | 570.31 | 538.71 | 200.21 | 137.80 | 540.69 | 436.80 |
| Mean | 468.72 | 385.89 | 599.63 | 549.27 | 215.15 | 174.32 | 463.12 | 407.33 |
| Standard deviations | 41.54 | 47.30 | 56.29 | 56.70 | 40.92 | 33.36 | 51.99 | 43.39 |
| 72h Repeat 1 | 859.23 | 666.76 | 1019.60 | 857.07 | 453.16 | 301.88 | 828.96 | 681.36 |
| Repeat 2 | 861.61 | 611.62 | 1060.38 | 978.89 | 396.15 | 346.11 | 927.73 | 747.48 |
| Repeat 3 | 952.28 | 748.65 | 998.43 | 1047.92 | 482.87 | 302.68 | 814.51 | 792.09 |
| Repeat 4 | 804.35 | 815.95 | 1011.76 | 921.22 | 567.99 | 276.14 | 843.42 | 754.65 |
| Repeat 5 | 814.69 | 714.60 | 1072.92 | 1055.23 | 506.96 | 324.40 | 865.10 | 729.16 |
| Mean | 858.43 | 711.51 | 1032.62 | 972.06 | 481.42 | 310.24 | 855.94 | 740.95 |
| Standard deviations | 58.431 | 77.90 | 32.28 | 84.45 | 63.68 | 26.35 | 44.27 | 40.42 |
| 96h Repeat 1 | 935.58 | 576.76 | 1202.30 | 1045.48 | 555.140 | 344.504 | 939.78 | 702.87 |
| Repeat 2 | 742.31 | 669.19 | 1330.9 | 1207.09 | 644.27 | 278.55 | 894.00 | 491.77 |
| Repeat 3 | 1083.51 | 541.89 | 1485.36 | 920.41 | 368.04 | 250.40 | 737.42 | 809.61 |
| Repeat 4 | 887.86 | 619.73 | 1299.53 | 1282.62 | 437.90 | 416.09 | 1032.12 | 639.14 |
| Repeat 5 | 969.78 | 852.43 | 1404.60 | 1125.07 | 492.51 | 379.89 | 967.08 | 729.16 |
| Mean | 923.81 | 652.00 | 1344.54 | 1116.13 | 499.57 | 333.89 | 914.08 | 674.51 |
| Standard deviations | 124.49 | 121.75 | 107.10 | 140.86 | 106.28 | 68.95 | 110.70 | 119.05 |

**Table-Fig 5B Raw data for Fig 5B**

|  | NC siRNA | Nrf2 siRNA | NC siRNA+  BMSCs-Exos | Nrf2 siRNA+  BMSCs-Exos | NC siRNA  +H_2_O_2_ | Nrf2 siRNA  +H_2_O_2_ | NC siRNA+  BMSCs-Exos+H_2_O_2_ | Nrf2 siRNA+  BMSCs-Exos+H_2_O_2_ |
| --- | --- | --- | --- | --- | --- | --- | --- | --- |
| Repeat 1 | 935.58 | 576.76 | 1202.30 | 1045.48 | 555.14 | 344.50 | 939.78 | 702.87 |
| Repeat 2 | 742.31 | 669.19 | 1330.89 | 1207.09 | 644.27 | 278.55 | 894.00 | 491.77 |
| Repeat 3 | 1083.51 | 541.89 | 1485.36 | 920.41 | 368.04 | 250.40 | 737.42 | 809.61 |
| Repeat 4 | 887.86 | 619.73 | 1299.53 | 1282.62 | 437.90 | 416.09 | 1032.12 | 639.14 |
| Repeat 5 | 969.78 | 852.43 | 1404.60 | 1125.07 | 492.51 | 379.89 | 967.08 | 729.16 |
| Mean | 923.81 | 652.00 | 1344.54 | 1116.13 | 499.57 | 333.89 | 914.08 | 674.51 |
| Standard deviations | 124.49 | 121.75 | 107.10 | 140.86 | 106.28 | 68.95 | 110.70 | 119.05 |

**Table-Fig 5C Raw data for Fig 5C**

|  | NC siRNA | Nrf2 siRNA | NC siRNA+  BMSCs-Exos | Nrf2 siRNA+  BMSCs-Exos | NC siRNA  +H_2_O_2_ | Nrf2 siRNA  +H_2_O_2_ | NC siRNA+  BMSCs-Exos+H_2_O_2_ | Nrf2 siRNA+  BMSCs-Exos+H_2_O_2_ |
| --- | --- | --- | --- | --- | --- | --- | --- | --- |
| Repeat 1 | 9.25 | 11.31 | 3.71 | 6.72 | 22.94 | 28.73 | 9.34 | 15.12 |
| Repeat 2 | 8.86 | 11.97 | 3.90 | 6.69 | 21.16 | 28.52 | 9.01 | 15.60 |
| Repeat 3 | 9.29 | 10.75 | 3.72 | 6.39 | 22.88 | 28.07 | 9.68 | 15.65 |
| Mean | 9.14 | 11.34 | 3.78 | 6.60 | 22.33 | 28.44 | 9.34 | 15.45 |
| Standard deviations | 0.24 | 0.61 | 0.11 | 0.19 | 1.00 | 0.34 | 0.34 | 0.29 |

**Table-Fig 5D Raw data for Fig 5D**

|  | NC siRNA | Nrf2 siRNA | NC siRNA+  BMSCs-Exos | Nrf2 siRNA+  BMSCs-Exos | NC siRNA  +H_2_O_2_ | Nrf2 siRNA  +H_2_O_2_ | NC siRNA+  BMSCs-Exos+H_2_O_2_ | Nrf2 siRNA+  BMSCs-Exos+H_2_O_2_ |
| --- | --- | --- | --- | --- | --- | --- | --- | --- |
| Repeat 1 | 5.16 | 4.19 | 12.01 | 7.95 | 2.74 | 1.85 | 5.53 | 4.28 |
| Repeat 2 | 6.14 | 5.32 | 12.40 | 10.05 | 3.45 | 1.84 | 6.83 | 4.93 |
| Repeat 3 | 6.04 | 4.22 | 10.19 | 8.10 | 2.52 | 1.62 | 5.08 | 4.78 |
| Mean | 5.78 | 4.58 | 11.53 | 8.70 | 2.90 | 1.77 | 5.81 | 4.66 |
| Standard deviations | 0.54 | 0.64 | 1.18 | 1.17 | 0.49 | 0.13 | 0.91 | 0.34 |

**Table-Fig 5E Raw data for Fig 5E**

|  | NC siRNA | Nrf2 siRNA | NC siRNA+  BMSCs-Exos | Nrf2 siRNA+  BMSCs-Exos | NC siRNA  +H_2_O_2_ | Nrf2 siRNA  +H_2_O_2_ | NC siRNA+  BMSCs-Exos+H_2_O_2_ | Nrf2 siRNA+  BMSCs-Exos+H_2_O_2_ |
| --- | --- | --- | --- | --- | --- | --- | --- | --- |
| Repeat 1 | 1.44 | 1.89 | 0.99 | 1.3 | 3.42 | 4.28 | 1.94 | 2.4 |
| Repeat 2 | 1.7 | 1.99 | 0.87 | 1.32 | 3.62 | 4.42 | 1.65 | 2.72 |
| Repeat 3 | 1.97 | 2.26 | 0.66 | 1.53 | 3.19 | 4.11 | 1.56 | 2.61 |
| Mean | 1.70 | 2.05 | 0.84 | 1.38 | 3.41 | 4.27 | 1.72 | 2.58 |
| Standard deviations | 0.27 | 0.19 | 0.17 | 0.16 | 0.16 | 0.12 | 0.27 | 0.15 |

**Table-Fig 5F Raw data for Fig 5F**

|  | NC siRNA | Nrf2 siRNA | NC siRNA+  BMSCs-Exos | Nrf2 siRNA+  BMSCs-Exos | NC siRNA  +H_2_O_2_ | Nrf2 siRNA  +H_2_O_2_ | NC siRNA+  BMSCs-Exos+H_2_O_2_ | Nrf2 siRNA+  BMSCs-Exos+H_2_O_2_ |
| --- | --- | --- | --- | --- | --- | --- | --- | --- |
| Repeat 1 | 3.32 | 2.7 | 7.02 | 5.23 | 1.63 | 1.11 | 3.54 | 2.72 |
| Repeat 2 | 3.36 | 2.54 | 6.57 | 4.87 | 1.87 | 0.85 | 3.31 | 2.84 |
| Repeat 3 | 3.47 | 2.81 | 6.72 | 5.12 | 1.67 | 1.05 | 3.35 | 2.62 |
| Mean | 3.38 | 2.68 | 6.77 | 5.07 | 1.72 | 1.00 | 3.40 | 2.73 |
| Standard deviations | 0.0777 | 0.14 | 0.23 | 0.18 | 0.13 | 0.14 | 0.12 | 0.11 |

**Table-Fig 6 Raw data for Fig 6**

|  |  | NC siRNA | NRF2 siRNA | NC siRNA+  BMSCs-Exos | NRF2 siRNA+  BMSCs-Exos | NC siRNA  +H_2_O_2_ | NRF2 siRNA  +H_2_O_2_ | NC siRNA+  BMSCs-Exos+H_2_O_2_ | NRF2 siRNA+  BMSCs-Exos+H_2_O_2_ |
| --- | --- | --- | --- | --- | --- | --- | --- | --- | --- |
| KI67 | Repeat 1 | 0.98 | 0.82 | 1.51 | 1.48 | 0.82 | 0.59 | 1.04 | 0.63 |
|  | Repeat 2 | 1.01 | 0.82 | 1.58 | 1.49 | 0.81 | 0.57 | 1.00 | 0.7 |
|  | Repeat 3 | 1.01 | 0.85 | 1.57 | 1.5 | 0.82 | 0.56 | 1.01 | 0.65 |
|  | Mean | 1.00 | 0.83 | 1.55 | 1.49 | 0.82 | 0.573333 | 1.02 | 0.66 |
|  | Standard deviations | 0.017 | 0.0173 | 0.0379 | 0.01 | 0.00577 | 0.0153 | 0.0208 | 0.036 |
| NRF2 | Repeat 1 | 0.99 | 1.01 | 2.79 | 1.82 | 0.89 | 0.40 | 1.12 | 0.56 |
|  | Repeat 2 | 1.04 | 1.02 | 2.74 | 1.72 | 0.95 | 0.38 | 1.07 | 0.60 |
|  | Repeat 3 | 0.97 | 0.99 | 2.75 | 1.76 | 0.90 | 0.38 | 1.10 | 0.59 |
|  | Mean | 1.00 | 1.01 | 2.76 | 1.77 | 0.91 | 0.387 | 1.10 | 0.58 |
|  | Standard deviations | 0.036055513.00 | 0.0153 | 0.0264 | 0.0503 | 0.0321 | 0.0115 | 0.0252 | 0.0208 |
| HO1 | Repeat 1 | 1.03 | 0.16 | 1.56 | 1.08 | 0.38 | 0.08 | 1.57 | 0.94 |
|  | Repeat 2 | 0.99 | 0.17 | 1.59 | 1.12 | 0.40 | 0.07 | 1.53 | 0.89 |
|  | Repeat 3 | 0.98 | 0.17 | 1.62 | 1.15 | 0.40 | 0.07 | 1.54 | 0.89 |
|  | Mean | 1.00 | 0.167 | 1.59 | 1.12 | 0.39 | 0.0733 | 1.55 | 0.91 |
|  | Standard deviations | 0.026 | 0.00577 | 0.03 | 0.035 | 0.0115 | 0.0057 | 0.0208 | 0.029 |
| BAX | Repeat 1 | 0.99 | 1.21 | 0.40 | 0.73 | 1.49 | 1.99 | 1.19 | 1.27 |
|  | Repeat 2 | 1.02 | 1.22 | 0.40 | 0.74 | 1.48 | 2.02 | 1.19 | 1.3 |
|  | Repeat 3 | 0.98 | 1.24 | 0.39 | 0.74 | 1.46 | 2.03 | 1.16 | 1.32 |
|  | Mean | 1.00 | 1.22 | 0.40 | 0.74 | 1.48 | 2.01 | 1.18 | 1.30 |
|  | Standard deviations | 0.021 | 0.0153 | 0.00577 | 0.00577 | 0.0153 | 0.021 | 0.0173 | 0.0252 |
| BCL-2 | Repeat 1 | 1.00 | 0.53 | 1.54 | 1.23 | 0.36 | 0.24 | 1.13 | 0.87 |
|  | Repeat 2 | 0.99 | 0.54 | 1.52 | 1.22 | 0.37 | 0.25 | 1.11 | 0.87 |
|  | Repeat 3 | 1.01 | 0.54 | 1.52 | 1.22 | 0.36 | 0.25 | 1.11 | 0.84 |
|  | Mean | 1.00 | 0.54 | 1.53 | 1.22 | 0.36 | 0.25 | 1.12 | 0.86 |
|  | Standard deviations | 0.01 | 0.00577 | 0.011 | 0.00577 | 0.00577 | 0.00577 | 0.0115 | 0.0173 |
