# Supplementary material for: Exosomes from bone marrow mesenchymal stem cells protect melanocytes under vitiligo-related conditions through induction of NRF2/HO1 expression
Source: PLoS One. 2025 Dec 4;20(12):e0338323. doi: 10.1371/journal.pone.0338323 (PMC12677447; doi:10.1371/journal.pone.0338323)
Supplement: Attachment — Submitted filename: Response to Reviewers.docx [file pone.0338323.s006.docx]

Dear Editor,

Thank you very much for reviewing our manuscript. We have carefully addressed all the issues raised by the editor and reviewers point by point, and have thoroughly revised the manuscript accordingly. These revisions are detailed in the uploaded files “Response to Reviewers,” “Revised Manuscript with Track Changes,” and “Manuscript.”. In addition, we have uploaded the raw data for the line and column charts (Figures 2-6) and the original, uncropped, and unadjusted Western blot images for Figures 4, 6, and S1 as Supporting Information files. All relevant data are included within the manuscript and its Supporting Information files. We confirm that this submission contains all raw data required to replicate the results of our study.

This work was supported by the Medicine and Hygiene Program of Zhejiang Province, Youth Innovation Project (No. 2023RC064). The funders had no role in study design, data collection and analysis, decision to publish, or preparation of the manuscript.

Should there be any questions, please do not hesitate to contact us.

Best regards

Aie Xu

Journal Requirements:

When submitting your revision, we need you to address these additional requirements.

1. Please ensure that your manuscript meets PLOS ONE's style requirements, including those for file naming. The PLOS ONE style templates can be found at

https://journals.plos.org/plosone/s/file?id=wjVg/PLOSOne_formatting_sample_main_body.pdf and

<https://journals.plos.org/plosone/s/file?id=ba62/PLOSOne_formatting_sample_title_authors_affiliations.pdf>.

2. Thank you for stating the following financial disclosure:

“The Medicine and Hygiene Program of Zhejiang Province, Youth Innovation Project (No. 2023RC064)”

Please state what role the funders took in the study.  If the funders had no role, please state: "The funders had no role in study design, data collection and analysis, decision to publish, or preparation of the manuscript."

If this statement is not correct you must amend it as needed.

Please include this amended Role of Funder statement in your cover letter; we will change the online submission form on your behalf.

3. We note that your Data Availability Statement is currently as follows: [All relevant data are within the manuscript and its Supporting Information files.

Please confirm at this time whether or not your submission contains all raw data required to replicate the results of your study. Authors must share the “minimal data set” for their submission. PLOS defines the minimal data set to consist of the data required to replicate all study findings reported in the article, as well as related metadata and methods (https://journals.plos.org/plosone/s/data-availability#loc-minimal-data-set-definition).

For example, authors should submit the following data:

- The values behind the means, standard deviations and other measures reported;

- The values used to build graphs;

- The points extracted from images for analysis.

Authors do not need to submit their entire data set if only a portion of the data was used in the reported study.

If your submission does not contain these data, please either upload them as Supporting Information files or deposit them to a stable, public repository and provide us with the relevant URLs, DOIs, or accession numbers. For a list of recommended repositories, please see https://journals.plos.org/plosone/s/recommended-repositories.

If there are ethical or legal restrictions on sharing a de-identified data set, please explain them in detail (e.g., data contain potentially sensitive information, data are owned by a third-party organization, etc.) and who has imposed them (e.g., an ethics committee). Please also provide contact information for a data access committee, ethics committee, or other institutional body to which data requests may be sent. If data are owned by a third party, please indicate how others may request data access.

***Response: Thank you for the suggestion. We have uploaded the raw data for the line and column charts (Figures 2-6) as a Supporting Information file.***

4. PLOS ONE now requires that authors provide the original uncropped and unadjusted images underlying all blot or gel results reported in a submission’s figures or Supporting Information files. This policy and the journal’s other requirements for blot/gel reporting and figure preparation are described in detail at https://journals.plos.org/plosone/s/figures#loc-blot-and-gel-reporting-requirements and https://journals.plos.org/plosone/s/figures#loc-preparing-figures-from-image-files. When you submit your revised manuscript, please ensure that your figures adhere fully to these guidelines and provide the original underlying images for all blot or gel data reported in your submission. See the following link for instructions on providing the original image data: https://journals.plos.org/plosone/s/figures#loc-original-images-for-blots-and-gels.

In your cover letter, please note whether your blot/gel image data are in Supporting Information or posted at a public data repository, provide the repository URL if relevant, and provide specific details as to which raw blot/gel images, if any, are not available. Email us at plosone@plos.org if you have any questions.

***Response: As suggested, we uploaded the original, uncropped, and unadjusted Western blot images for Figures 4, 6, and S1 as Supporting Information files. Thank you.***

5. PLOS requires an ORCID iD for the corresponding author in Editorial Manager on papers submitted after December 6th, 2016. Please ensure that you have an ORCID iD and that it is validated in Editorial Manager. To do this, go to ‘Update my Information’ (in the upper left-hand corner of the main menu), and click on the Fetch/Validate link next to the ORCID field. This will take you to the ORCID site and allow you to create a new iD or authenticate a pre-existing iD in Editorial Manager.

***Response: The ORCID ID of the corresponding author has been linked to the submission account. Thank you for your kind reminder.***

6. Thank you for stating the following in the Acknowledgments Section of your manuscript:

” This work was supported by the Medicine and Hygiene Program of Zhejiang Province, Youth Innovation Project (No. 2023RC064).”

We note that you have provided additional information within the Acknowledgements Section that is not currently declared in your Funding Statement. Please note that funding information should not appear in the Acknowledgments section or other areas of your manuscript. We will only publish funding information present in the Funding Statement section of the online submission form.

Please remove any funding-related text from the manuscript and let us know how you would like to update your Funding Statement. Currently, your Funding Statement reads as follows:

“The Medicine and Hygiene Program of Zhejiang Province, Youth Innovation Project (No. 2023RC064)”

Please include your amended statements within your cover letter; we will change the online submission form on your behalf.

***Response: Thank you for the suggestion. We have removed the funding description from the manuscript and added a funding statement in the Response to Reviewers as follows: This work was supported by the Medicine and Hygiene Program of Zhejiang Province, Youth Innovation Project (No. 2023RC064). The funders had no role in study design, data collection and analysis, decision to publish, or preparation of the manuscript.***

7. If the reviewer comments include a recommendation to cite specific previously published works, please review and evaluate these publications to determine whether they are relevant and should be cited. There is no requirement to cite these works unless the editor has indicated otherwise. 

Additional Editor Comments (if provided):

The manuscript "Exosomes from bone marrow mesenchymal stem cells protect melanocytes under vitiligo-related conditions through induction of NRF2/HO1 expression" is interesting; however, as reviewers stated, it needs to be revised according to their suggestions. Therefore, it is advisable to revise the manuscript in light of the reviewers' comments.


[Note: HTML markup is below. Please do not edit.]

Reviewers' comments:

Reviewer's Responses to Questions

**Comments to the Author**

1. Is the manuscript technically sound, and do the data support the conclusions?

The manuscript must describe a technically sound piece of scientific research with data that supports the conclusions. Experiments must have been conducted rigorously, with appropriate controls, replication, and sample sizes. The conclusions must be drawn appropriately based on the data presented.

Reviewer #1: Yes

Reviewer #2: Partly

2. Has the statistical analysis been performed appropriately and rigorously?

Reviewer #1: Yes

Reviewer #2: Yes

3. Have the authors made all data underlying the findings in their manuscript fully available?

The [PLOS Data policy](http://www.plosone.org/static/policies.action#sharing) requires authors to make all data underlying the findings described in their manuscript fully available without restriction, with rare exception (please refer to the Data Availability Statement in the manuscript PDF file). The data should be provided as part of the manuscript or its supporting information, or deposited to a public repository. For example, in addition to summary statistics, the data points behind means, medians and variance measures should be available. If there are restrictions on publicly sharing data—e.g. participant privacy or use of data from a third party—those must be specified.

Reviewer #1: Yes

Reviewer #2: Yes

4. Is the manuscript presented in an intelligible fashion and written in standard English?

PLOS ONE does not copyedit accepted manuscripts, so the language in submitted articles must be clear, correct, and unambiguous. Any typographical or grammatical errors should be corrected at revision, so please note any specific errors here.

Reviewer #1: Yes

Reviewer #2: No

5. Review Comments to the Author

Please use the space provided to explain your answers to the questions above. You may also include additional comments for the author, including concerns about dual publication, research ethics, or publication ethics. (Please upload your review as an attachment if it exceeds 20,000 characters)

Reviewer #1: The manuscript is of good understanding towards the vitiligo and has potential to be carried in geenrating the therapeutic applications. Its well written and informed.
For improvement, manuscript may also provide details of the MSCs characterization as no detail on surface markers is given.

***Response: We appreciate the reviewer’s comment. BMSCs are characterized by the expression of surface markers CD105, CD44, CD29, CD73, and CD90, while lacking hematopoietic and myeloid markers such as CD34, CD45, and CD11b [15, 16]. In our study, BMSCs were obtained from Cyagen Biosciences (Guangzhou, China) and were positive for CD29, CD44, and CD90, but negative for CD34, CD45, CD11b. This information has been added to the subsection BMSCs culturing and identification (Page 5).***

***References:***

***15. Maličev E, Jazbec K. An Overview of Mesenchymal Stem Cell Heterogeneity and Concentration. Pharmaceuticals (Basel). 2024 Mar 7;17(3):350. doi: 10.3390/ph17030350. PMID: 38543135.***

***16. Gao Q, Wang L, Wang S, Huang B, Jing Y, Su J. Bone Marrow Mesenchymal Stromal Cells: Identification, Classification, and Differentiation. Front Cell Dev Biol. 2022 Jan 3;9:787118. doi: 10.3389/fcell.2021.787118. PMID: 35047499.***

Further, details about the role of each agent used in the vitiligo study and its therapeutic effect with the exosomes may be given.

***Response: We appreciate the reviewer’s valuable comment. We added a description of the role of Keratinocyte-SFM in vitiligo study in the subsection Culturing and treatment of PIG3V cells: “Keratinocyte-SFM is specifically formulated to support the growth and maintenance of human keratinocytes (such as PIG3V cells) under serum-free conditions, though it is sometimes supplemented with FBS and antibiotics (e.g., penicillin-streptomycin) to enhance cell support and prevent contamination” (Page 7-8).***

***The roles of GW4869 and H₂O₂ , which were applied to PIG3V cells, have already been addressed in the same subsection: “conditioned medium from BMSCs pretreated with GW4869 (an inhibitor of exosome release) at a final concentration of 5 µM (referred to as BMSCs-GW4869)”, and “Treatments were applied either under normal conditions or following hydrogen peroxide (H₂O₂) exposure to mimic vitiligo-related oxidative stress in vitro” (Page 8)***

Reviewer #2: Title: Exosomes from bone marrow mesenchymal stem cells protect melanocytes under vitiligo-related condition through induction of NRF2/HO1 expression

This is a well-designed and timely study that investigates a promising cell-free therapeutic strategy for vitiligo. The research question is clear and clinically relevant. The manuscript provides compelling in vitro evidence that exosomes derived from Bone Marrow Mesenchymal Stem Cells (BMSCs-Exos) can protect melanocytes from oxidative stress—a key driver of vitiligo pathogenesis—by activating the NRF2/HO1 antioxidant pathway. The experimental design is logical, and the data generally support the conclusions. The findings could have significant implications for developing novel treatments for vitiligo.
***Response: Thanks for these positive comments.***

Strengths
1. The study addresses a significant unmet need in vitiligo treatment by exploring BMSCs-Exos as a safer, "off-the-shelf" alternative to whole MSC transplantation, mitigating risks like low engraftment survival and potential tumorigenicity.

2.The authors thoroughly evaluated the protective effects of BMSCs-Exos by assessing multiple endpoints: cell viability, apoptosis, and a panel of oxidative stress markers (GSH/GSSG, SOD, ROS, MDA). This multi-faceted approach strengthens the conclusions.

3. The use of conditioned medium from BMSCs treated with the exosome inhibitor GW4869 (BMSCs-GW4869) is a critical and well-considered control. It helps to attribute the observed effects specifically to the exosomes themselves rather than other soluble factors secreted by BMSCs.

4. The use of siRNA to knockdown *NRF2* expression is the strongest part of the manuscript. It effectively demonstrates that the protective effects of BMSCs-Exos are dependent on the NRF2/HO1 pathway, providing causal evidence for the proposed mechanism.
***Response: Thanks for the above positive comments.***

Shortcomings
1.The authors note that BMSCs-GW4869 still had a significant effect on apoptosis (Fig 2C), which they correctly attribute to potentially incomplete inhibition of exosome release. This is a limitation. The manuscript would be strengthened by including data (e.g., NTA or Western blot of markers) showing the actual reduction in exosome yield in the GW4869-conditioned medium to quantify the efficacy of inhibition.

***Response: We appreciate the reviewer’s valuable comment. Our result showed that BMSCs-GW4869 mitigated the effect of BMSCs-conditioned*** ***medium in most experiments; however, in Fig 2C, both BMSCs-medium and BMSCs-GW4869 decreased apoptosis in H₂O₂-treated PIG3V cells. We speculate that this might be due to the used 5 µM dose of GW4849 being insufficient to fully suppress exosome release in BMSCs-conditioned medium. We acknowledge that quantifying exosomes via NTA, exosome marker measurement, etc. would help confirm the efficacy of inhibition. Unfortunately, due to missing samples and limited research funding, it was difficult for us to conduct these supplementary experiments. We have further discussed this in the Discussion section:***

***“Although BMSCs-GW4869 mitigated the effect of BMSCs-medium, in some results it also significantly affected the viability and apoptosis of PIG3V cells. This***

***probably could be due to inadequately suppression of release of exosomes from BMSCs into the conditioned medium at the relatively low concentration (5 μM) of GW4869 used in this study. In future studies, exosomes will be quantified using NTA, exosome marker measurement, etc. to determine the inhibition efficacy of GW4869 in the release of exosomes. Moreover, more appropriate dosage of GW4869 in combination with silencing of exosome activity-related genes will be employed to more effectively inhibit exosomes released from MSCs to further validate the role of BMSCs-Exos in vitiligo.” (The* ***last paragraph of Page 17*** *and the* ***first paragraph of Page 18****)***

2. The entire study is conducted in an in vitro cell line model (PIG3V). While this is a valid starting point, the conclusions about therapeutic potential for vitiligo would be significantly more powerful if supported by data from an in vivo animal model of vitiligo
***Response: We appreciate the reviewer’s constructive comment. This study is an in vitro investigation, demonstrating that BMSCs-Exos protect melanocytes from vitiliog-related H₂O₂-induced oxidative stress through induction of NRF2/HO1 expression. We agree that the conclusion would be further strengthened by in vivo experiments. Nevertheless, constrained by time and research funding, these experiments could not be completed within the current study period. In future work, a mouse model of vitiligo will be established and used to validate these findings in vivo. This has been addressed in the Discussion section. (Page 19)***

3.The study identifies the NRF2/HO1 pathway as the mechanism but does not investigate what component of the BMSCs-Exos is responsible for activating this pathway. Is it specific miRNAs, proteins, lipids, or mRNAs? Identifying the active cargo would be a major advance and is a natural next step.
***Response: We appreciate this valuable suggestion. Identifying the specific components of BMSCs-Exos (such as mRNA, noncoding RNAs, miRNAs, mRNAs, and proteins) that regulate NRF2/HO1 signaling to protect H_2_O_2_-treated PIG3V cells would really be a significant advancement. To further elucidate the related molecular mechanisms, transcriptomic, proteomic and metabolomic analyses will be conducted to screen differentially expressed miRNAs, mRNAs, proteins, etc. Subsequently, exosomes carrying target mRNA/shRNA, miRNA minic/inhibitor, etc. will be generated through genetic manipulation of BMSCs and used for functional validation in vitro and in vivo. However, these experiments, which constitute a series of studies, are expected to take at least one year and could not be completed within the current study frame. This has been discussed in the Discussion section:***

***“This study did not identify the specific components of BMSCs-Exos (such as mRNA, noncoding RNAs, miRNAs, mRNAs, and proteins) that regulate NRF2/HO1 signaling to protect H_2_O_2_-treated PIG3V cells. In future studies, transcriptomic, proteomic and metabolomic analyses combined with in vitro and in vivo functional validation will be conducted. These investigations will focus on generating exosomes with altered expression of target molecules to identify upstream effectors (such as mRNAs, noncoding RNAs, miRNAs, and proteins) within BMSCs-Exos that activate the NRF2/HO1 signaling, thereby further elucidating the molecular mechanisms underlying the protective role of BMSCs-Exos in vitiligo.” (Page 19).***

4.The findings are based on a single immortalized human melanocyte cell line (PIG3V). Repeating key experiments in primary human melanocytes would enhance the translational relevance and generalizability of the results.
***Response: Thank you for the valuable suggestion. As mentioned in Introduction, the effect and underlying mechanisms of MSCs-derived exosomes in vitiligo remain unclear. Therefore, we carried out a pioneering in vitro study demonstrating that BMSCs-Exos protect melanocytes exposed to vitiliog-related H₂O₂-induced oxidative stress by induction of NRF2/HO1 expression. Compared with immortalized human melanocyte cell lines, primary human melanocytes more closely resemble in vivo physiological conditions, providing results with greater translational relevance and and generalizability. However, the isolation and culture of primary human melanocytes require hospital ethical approval and considerable time. Therefore, in the current study, we used the human melanocyte cell line PIG3V. Due to limited time and research funding, it was not feasible for us to repeat key experiments using primary human melanocytes. We have added relevant discussion in the Discussion section:***

***“We carried out a pioneering in vitro study to investigate the effect and underlying mechanisms of MSCs-derived exosomes in vitiligo. Compared with immortalized human melanocyte cell lines, primary human melanocytes more closely resemble in vivo physiological conditions, providing results with greater translational relevance and generalizability. However, the isolation and culture of primary human melanocytes require hospital ethical approval and considerable time. Therefore, in the current study, we used the human melanocyte cell line PIG3V. To enhance the translational value of the findings, primary human melanocytes will be isolated, cultured, and used in future studies to validate the results obtained from PIG3V cells.” (The* ***last paragraph of Page 18****)***

5. The exosome inhibitor is consistently misspelled. It is GW4869, not "GW4849" (e.g., Abstract, Methods, Results, Figure legends).
***Response: Thank you for pointing this out. "GW4849" has been corrected to"GW4869" throughout the manuscript. Thanks.***

6. Page 23, Line 3: "BMCs-Exos" should be "BMSCs-Exos".
***Response: Thank you for the careful review. "BMCs-Exos" has been revised to "BMSCs-Exos" in the manuscript.***

7. Page 24, Line 1: "Bcl-X" is likely a typo and should be "BAX".
***Response: Thank you for pointing this out. "Bcl-X" was a typo and has been corrected to "BAX" in the manuscript.***

8. Page 25, Line 3: "NO-1" is a typo and should be "HO1".
***Response: Thank you for noting this. "NO-1" is a typo and has been revised to "HO1" in the manuscript.***

9.The term "BMSCs-GW4849" is used in the text, but the figures (e.g., Fig 2, 3, 4) use the correct "BMSCs-GW4869". This must be harmonized.
***Response:* Thank you for noting this inconsistency. "*GW4849" in the text has been corrected to "GW4869" throughout to ensure consistency with the figures.***

10.The figure legends are very long and seemingly complicated, making them difficult to follow. The descriptions of each experimental group could be summarized in a table format within the legend or in a supplementary table for clarity.
***Response: Thank you for the suggestion. The descriptions of each experimental group have been summarized in supplementary tables to improve clarity***

11.The statistical annotation in Figure 5 is particularly complex and hard to decipher (e.g., `[5]`, `[55]`, `[&]`, `&&&`). Using more standard notations (e.g., asterisks and hashtags with clear definitions) would greatly improve readability.
***Response: Thank you for pointing this out. The statistical annotations in Figures 5 and 6 have been revised using more standard notations with clear definitions provided in the legend to improve readability.***

12.In the Supplementary Figure legend (Page 35), it says "Fig 1B", "Fig 1C", etc. It should be "Fig S1B", "Fig S1C" for consistency.
***Response: Thank you for noting this. “Fig 1B”, “Fig 1C”, etc. in the Supplementary Figure legend have been corrected to “Fig S1B”, “Fig S1C”, etc. for consistency.***

13.While generally understandable, the manuscript would benefit from thorough proofreading by a native English speaker or a professional editing service to improve fluency. Some examples:
14.Page 11: "...causing white skin patches and reduced quality of life" -> "...causing depigmented patches on the skin and a reduced quality of life".
15.Page 12: "MSCs exhibit capacities of extensive proliferation..." -> "MSCs have a high capacity for extensive proliferation..."
16.Page 13: "Exosomes are safe without risk of tumorigenicity..." -> "Exosomes are safe, with no risk of tumorigenicity..."

***Response: Thank you for the suggestion. We have thoroughly reviewed and improved the English language throughout the manuscript.***

6. PLOS authors have the option to publish the peer review history of their article ([what does this mean?](https://journals.plos.org/plosone/s/editorial-and-peer-review-process#loc-peer-review-history)). If published, this will include your full peer review and any attached files.
   ***Response: Thank you for noting this. The peer review history is available for reference.***
